# Growth-uncoupled propanediol production in a *Thermoanaerobacterium thermosaccharolyticum* strain engineered for high ethanol yield

**DOI:** 10.1038/s41598-023-29220-9

**Published:** 2023-02-10

**Authors:** Christopher D. Herring, Maulana Permana Ajie, Lee R. Lynd

**Affiliations:** 1 Terragia Biofuel Incorporated, Hanover, New Hampshire, United States; 2 Present Address: Technical University of Munich, Munich, Germany; 3 Dartmouth College, Thayer School of Engineering, Hanover, New Hampshire United States; 4 Center for Bioenergy Innovation, Oak Ridge, Tennessee United States; 5 Bioengineering, Rhine-Waal University of Applied Sciences, Kleve, Germany

**Keywords:** Industrial microbiology, Metabolic engineering

## Abstract

Cocultures of engineered thermophilic bacteria can ferment lignocellulose without costly pretreatment or added enzymes, an ability that can be exploited for low cost biofuel production from renewable feedstocks. The hemicellulose-fermenting species *Thermoanaerobacterium thermosaccharolyticum* was engineered for high ethanol yield, but we found that the strains switched from growth-coupled production of ethanol to growth uncoupled production of acetate and 1,2-propanediol upon growth cessation, producing up to 6.7 g/L 1,2-propanediol from 60 g/L cellobiose. The unique capability of this species to make 1,2-propanediol from sugars was described decades ago, but the genes responsible were not identified. Here we deleted genes encoding methylglyoxal reductase, methylglyoxal synthase and glycerol dehydrogenase. Deletion of the latter two genes eliminated propanediol production. To understand how carbon flux is redirected in this species, we hypothesized that high ATP levels during growth cessation downregulate the activity of alcohol and aldehyde dehydrogenase activities. Measurements with cell free extracts show approximately twofold and tenfold inhibition of these activities by 10 mM ATP, supporting the hypothesized mechanism of metabolic redirection. This result may have implications for efforts to direct and maximize flux through alcohol dehydrogenase in other species.

## Introduction

Technology for biofuel production from lignocellulose could have a large impact on reducing global carbon emissions, but a renewable biofuel industry failed to launch due to high costs associated with pretreatment and enzyme production^1–3^. Lignocellulose deconstructing bacteria represent a way to avoid those costs because they can solubilize lignocellulose without costly pretreatment and produce ethanol at high yield^4–6^. While there is a large market for cellulosic ethanol, an even larger market exists for longer chain hydrocarbons, which can be produced from ethanol catalytically at low cost^7^.

The major focus of work on thermophilic Consolidated Bioprocessing (CBP) has focused *Clostridium thermocellum* due to its ability to ferment cellulose, but it cannot ferment 5-carbon sugars, which comprise a substantial fraction of the available carbohydrate in lignocellulose. A binary coculture of *C. thermocellum* and a compatible hemicellulose-fermenting microbe is a promising approach^8^. *Thermoanaerobacterium saccharolyticum* ferments the hemicellulose component of lignocellulose, and has been engineered to produce up to 70 g/L ethanol^5^. However, it does not grow well or ferment xylan above pH 6.7, while the optimum for *C. thermocellum* is pH 6.7–7.0^9^. Repeated attempts by the author’s group to grow cocultures of *C. thermocellum* and *T. saccharolyticum* have not been satisfactory (unpublished data).

*T. thermosaccharolyticum* (previously known as *Clostridium thermosaccharolyticum*) is a similar hemicellulose-fermenting species with a pH optimum of 7.0 or higher^10,11^, and has long been recognized as a potential species of industrial utility. In 1981, strain HG-4 was grown with *C. thermocellum* to produce 25.3 g/L ethanol from model substrates and 9.7 g/L ethanol from corn stover^12^. Strain HG-4 was derived from strain HG-2, isolated as a contaminant in a stock of *C. thermocellum*^13^. Strain HG-8 used in the present study (designated LL1244 in our strain collection) was derived from HG-4 in a process of mutagenesis and selection for high ethanol yield (US Patent 4,568,644). In 1986, Cameron and Cooney described the production of 1,2-propanediol from sugars in this strain^14^, and its potential commercial application. More than 1 billion pounds of 1,2-propanediol are produced annually in the United States^15^, but fermentation from renewable feedstocks is not yet cost-competitive with its chemical synthesis^16^. Many bacteria, including *T. thermosaccharolyticum,* produce 1,2-propanediol from the sugar rhamnose^11,16^. Efforts were made to engineer 1,2-propanediol production from glucose in *E. coli*, with a maximum titer of 4.5 g/L^17^. Production in *T. thermosaccharolyticum* was optimized, increasing titer to 9.1 g/L, but those efforts were limited by the lack of genome sequence and genetic tools^18,19^.

The author’s laboratory has worked for many years with *T. thermosaccharolyticum* with the goal of maximizing its ethanol production^20,21^. When the need for a pH-compatible coculture partner for *C. thermocellum* became evident, we intended to apply to *T. thermosaccharolyticum* the same metabolic engineering methods that had been used successfully in *T. saccharolyticum* to generate a strain able to produce ethanol at high yield and titer. However, previous efforts to eliminate acetate production in *T. thermosaccharolyticum* via genetic engineering were unsuccessful (A.J. Shaw, personal communication). Butanol pathway genes could not be expressed in acetate-deficient strains^22^, so the difficulty was ascribed to interference with the butanol pathway, known to be present in *T. thermosaccharolyticum*. However, a genome sequence for *T. thermosaccharolyticum* strain HG-8 generated by JGI (GCA_024171655.1) shows that 5 out of 7 genes related to butanol in strain DSM571 are deleted in strain HG-8 (TTHE_RS08230, TTHE_RS08235, TTHE_RS08240, TTHE_RS08245, TTHE_RS08250). Therefore, some other factor must have been responsible for the difficulty.

*T. saccharolyticum* and *T. thermosaccharolyticum* are similar at the sequence level but show significant differences in gene content. Genomic comparison reveals that 34% of *T. saccharolyticum* predicted protein sequences match a protein in *T. thermosaccharolyticum* DSM571 with > 90% identity, and another 38% show > 50% identity, but 20% show no match. In the course of further work engineering high ethanol yield in *T. thermosaccharolyticum* HG-8, unexpected differences were encountered, requiring the investigation of propanediol formation. This work led to results with implications for the broader topic of redirecting carbon flux, reported here.

## Results and Discussion

### Genetic engineering for high ethanol yield in *T. thermosaccharolyticum*

A two-step procedure was used to delete genes for high ethanol yield in *T. thermosaccharolyticum* strain HG-8: 1) a kanamycin resistance gene for positive selection and a *tdk* (thymidine kinase) gene for negative selection were inserted into a gene of interest; 2) the insertion was replaced with the sequence of the desired markerless deletion using FUDR counterselection. Using this method, the *pta* (phosphotransacetylase) or *pta/ack* (acetate kinase) genes were deleted. Resulting strains did not produce acetate, but grew very slowly, even after extensive serial transfers (Table 1).

**Table 1:** Knockout strains derived from strain HG-8.

Strain designation	Genotype	Growth	Acetate	Lactate
LL1411	*tdk*	+	+	+
LL1429	*tdk pta::kan*	Slow	−	+
LL1450	*tdk pta*	Slow	−	+
LL1451	*tdk pta/ack*	Slow	−	+
LL1452	*tdk ldh::kan*	+	+	−
LL1453	*tdk pta ldh::kan*	Slow	−	+
LL1514	*tdk pta ldh*	Slow	−	+
LL1547	*tdk pta ldh rnf*	Slow	−	+
LL1548	*tdk ldh rnf hfsB*	+	Reduced^1^	−
LL1562	*tdk ldh rnf hfsB adhE G544A*	+	Reduced^1^	−
LL1571	*tdk ldh hfsB*	+	Reduced^1^	−

^1^Acetate nearly eliminated when batch cultures were carbon limited.

With some difficulty, an additional deletion was made of the *ldh* (L-lactate dehydrogenase) gene, but the strain still grew slowly, and persisted in making a product that appeared to be lactate (strain LL1514). In contrast, interruption of *ldh* when *pta/ack* was intact eliminated lactate production (strain LL1452). Since *ldh* and *pta/ack* deletions in *T. saccharolyticum* eliminate lactate and acetate production without a persistent growth defect^23^, some difference should therefore exist between the species that affects its response to those deletions. Genes for bifurcating lactate dehydrogenase (*lctD*) and for *rnf* are present in *T. thermosaccharolyticum* but not *T. saccharolyticum*, but deletion of those genes did not affect the phenotype (LL1547). An alternate explanation is that the peak observed on HPLC of LL1514 is not actually lactate but some other product with the same retention time. Supporting this possibility, the UV to RI ratio of the peak was much lower than that of either D- or L-lactate.

Rather than directly deleting *pta/ack*, an indirect strategy was used to eliminate acetate production^24,25^. Deletion of *hfsB* resulted in almost complete elimination of acetate production while retaining normal growth (strain LL1548), but acetate levels were dependent on the conditions tested. Initial tests were performed with low substrate levels and measured at early timepoints in batch culture. Additional bottle fermentations with *hfsB* strains (Table 2) showed that for initial xylose concentrations up to 20 g/L, acetate levels were low and ethanol was produced at close to theoretical yield. At 40 g/L initial xylose, acetate levels were higher, ethanol yield was lower and some residual xylose remained.

**Table 2:** Bottle fermentations with xylose, sampled at 34–36 h (concentrations given in g/L).

Strain	Initial xylose	5 g/L xylose		10 g/L xylose		20 g/L xylose		40 g/L xylose		
	Genotype	ethanol	acetate	ethanol	acetate	ethanol	acetate	ethanol	acetate	xylose
LL1548	*tdk ldh rnf hfsB*	2.68	0.37	5.18	0.14	9.84	0.05	12.43	0.74	9.29
LL1562	*tdk ldh rnf hfsB adhE-G544A*	2.26	0.33	4.49	0.30	9.29	0.16	17.12	0.33	0.16
LL1571	*tdk ldh hfsB*	2.92	0.40	5.66	0.13	10.19	0.06	6.73	1.43	18.46

Previous work with *T. saccharolyticum* encountered problems using monomer xylose or glucose concentrations above approximately 30 g/L, however maltodextrin and cellobiose were found to be better tolerated and bottle fermentations were conducted with up to 150 g/L^5^. In the present study, bottle fermentations were conducted with 80 g/L maltodextrin to explore the effects of media components on ethanol titer (Table 3). The largest increase was observed with the doubling of all medium components or by addition of a defined vitamin and amino acid mixture. The complete amino acid mixture was more stimulatory than sub-mixes or individual amino acids. The association of higher nutrients and ethanol titers suggested that ethanol may be growth coupled in strain HG-8.

**Table 3:** Bottle fermentation with 80 g/L maltodextrin and strain LL1562.

Medium	Ethanol g/L	S.D.	Acetate g/L	S.D.	n
1 × medium CC6	27.97	0.66	3.57	0.60	6
2 × medium CC6	36.88	0.16	4.60	0.01	2
CC6 + 5 g/L yeast extract	32.02	0.35	3.36	0.10	2
CC6 + AA + Vit	36.44	1.17	2.29	0.22	6
CC6 + AA Group A + Vit	31.76	0.32	2.39	0.13	2
CC6 + AA Group B + Vit	34.53	n/a	2.09	n/a	1
CC6 + Arg + Cys + His	33.26	0.63	2.50	0.18	2
CC6 + Lys + Met + Thr	33.71	1.20	1.98	0.20	4
CC6 + Lys	29.86	0.07	2.10	0.04	2
CC6 + Met	32.20	0.24	1.90	0.12	2
CC6 + Thr	30.65	0.39	2.15	0.05	2

AA = defined amino acid mixture; Vit. = vitamins (see Methods).

### Propanediol production

In another experiment seeking to produce high ethanol titers, a fed-batch fermentation was conducted with 100 g/L xylose. In 26 h, 27 g/L of xylose had been consumed, producing 14 g/L ethanol and 1 g/L acetate. However at 47 h, 69 g/L of xylose was consumed, producing 16 g/L ethanol and 5 g/L acetate. Examination of the HPLC chromatogram revealed an unfamiliar peak, which was determined to be 1,2-propanediol at a final concentration of 5.6 g/L.

**Figure 1:**
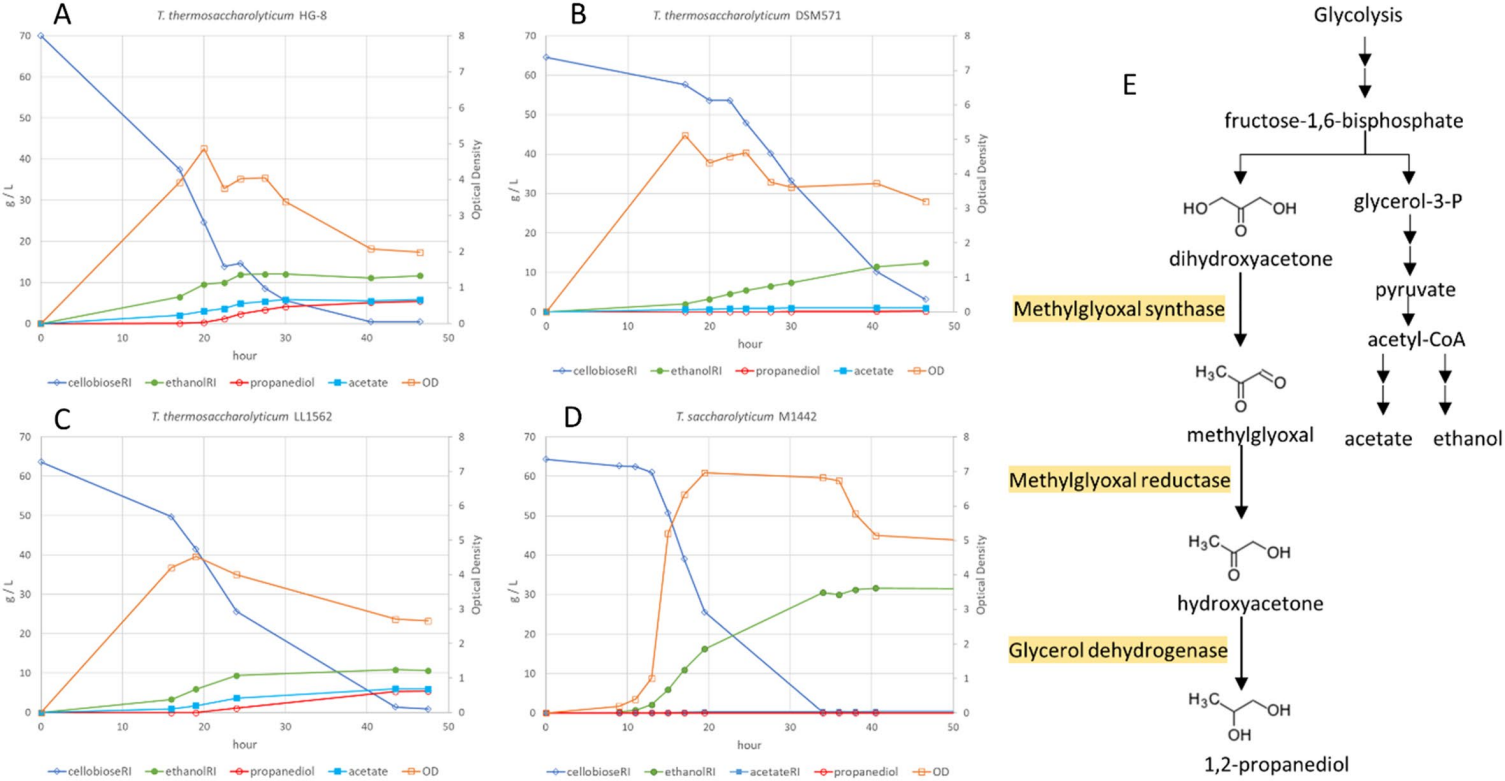
1,2-Propanediol production. (**A**–**D**) Fermentations of 60 g/L cellobiose with substrate and product concentrations shown on the left axes and optical density (OD) on the right axes. The strain used in each panel is indicated. Residual sugars (glucose and cellobiose) at the final time point were A: 5.4 g/L; B: 24.0 g/L; C: 11.1 g/L; D: 0.1 g/L. E: pathway for 1,2-propanediol proposed by Cameron and Cooney^14^.

To determine whether ethanol production in HG-8 is growth coupled, fermentations were conducted with 60 g/L cellobiose (Fig. 1 A-C). For *T. thermosaccharolyticum* HG-8, ethanol levels plateaued at 12 g/L shortly after the maximum optical density. Propanediol was not produced until the peak optical density, and continued to be produced up to 5.5 g/L after ethanol production stopped. Acetate was produced during growth (2 g/L), but also after (another 4 g/L), apparently in parallel to propanediol production. As noted by Cameron and Cooney^14^, production of a 1:1 ratio of propanediol and acetate is redox-balanced. Under the same conditions, *T. thermosaccharolyticum* strain DSM571 does not produce detectable propanediol and ethanol production continues in a growth-uncoupled fashion (Fig. 1B).

Propanediol production similar to that of strain HG-8 was observed for the high ethanol yielding strain LL1562 under the same conditions (Fig. 1C). Propanediol production started after maximum optical density, along with an increase in acetate production and a tapering of ethanol production. Glucose, produced by extracellular hydrolysis of cellobiose, accumulated to 10 g/L in this fermentation. In contrast, the high ethanol-yielding *T. saccharolyticum* strain M1442 grown under the same conditions (Fig. 1D) rapidly consumed all available sugar and produced 30 g/L ethanol, largely in a growth-uncoupled fashion. M1442 is a different species and was the subject of industrial strain development^5^, but nevertheless, the differences in performance are dramatic.

The association of propanediol and growth cessation has not been described previously. Production of 1,2-propanediol in strain HG-8 was independent of growth phase in the work of Sanchez-Riera et al.^18^, but was strongly associated with growth cessation in the phosphate-limited and other batch cultures of Hill et al.^20^. Phosphate levels affect 1,2-propanediol production in *C. sphenoides*^26^, and phosphate levels in our experiments are approximately fourfold lower than those used by Cameron et al., so medium differences might account for the differences in observed profiles.

### Genetic deletions to eliminate propanediol production

The enzymes responsible for production of 1,2-propanediol in *T. thermosaccharolyticum* were not determined by Cameron et al. due to the lack of genome sequence and genetic tools. However, a pathway was proposed based on the detection of hydroxyacetone (AKA acetol) in culture broth (Fig. 1E). The first step of this pathway, methylglyoxal synthase, is easily recognizable in the HG-8 genome (ORF_0295), and is also present in *T. thermosaccharolyticum* DSM571 and *T. saccharolyticum* JW/SL-YS485 (from which M1442 was derived). The second step, methylglyoxal reductase, is harder to identify due to the broad substrate specificity and multi-functionality of enzymes known to reduce methylglyoxal^27^. Two adjacent genes (ORF_2845 and ORF_2846) exist in the HG-8 genome with the annotation “aldo/keto reductase” that we hypothesize to encode methylglyoxal reductase, based on similarity to the *E. coli* methylglyoxal reductase gene *ydjG*. The third step, converting hydroxyacetone to 1,2-propanediol, is known to be catalyzed by glycerol dehydrogenase, but a clear assignment is difficult due to multiple activities of this enzyme, including converting dihydroxyacetone to glycerol as well as converting methylglyoxal to R-lactaldehyde. Glycerol dehydrogenase occurs in HG-8 (ORF_2448), but does not occur in *T. saccharolyticum*, consistent with the lack of propanediol production in *T. saccharolyticum*.

Ostensibly, some or all genes of the propanediol pathway should be present in HG-8 but not in *T. saccharolyticum*. The presence of methylglyoxal synthase but lack of glycerol dehydrogenase suggests that *T. saccharolyticum* may be able to use methylglyoxal as a glycolytic bypass returning the carbon to pyruvate, while *T. thermosaccharolyticum* HG-8 converts it to 1,2-propanediol as a terminal route. HG-8 was subject to mutagenesis and selection, potentially disrupting numerous genes. Deletions in the butanol acid pathway are one example. It is possible that the peculiar production of 1,2-propanediol in HG-8 is due to loss of some activity that would normally return carbon from methylglyoxal to glycolysis.

Deletions were made of all three hypothesized genes in both the wild type HG-8 and LL1562 ethanologen backgrounds, replacing the targeted genes with the kamanycin resistance/thymidine kinase cassette used previously. Bottle fermentations with maltodextrin (Table 4) showed significantly lower (P = 0.02) propanediol levels in all three knockouts in the ethanologen LL1562 background, close to the limit of detection. In the wild type background however, propanediol was undetectable for the methylglyoxal synthase and glycerol dehydrogenase knockouts, and higher than the parent for the methylglyoxal reductase knockout.

**Table 4:** Bottle fermentations with knockout strains, pH 6.2, buffered with CaCO_3_.

Maltodextrin g/L	Background	KO Strain	Gene deleted	1,2-propanediol g/L	Acetate g/L	Ethanol g/L	Lactate g/L
80	LL1562		none	0.53	3.72	19.49	0.04
80	LL1562	LL1724	MG synthase^1^	0.22	1.44	24.16	0.00
80	LL1562	LL1723	MG reductase^1^	0.25	1.59	24.40	0.00
80	LL1562	LL1725	Glycerol dehydrogenase	0.20	1.33	23.58	0.00
40	HG-8		none	1.08	3.45	8.46	0.79
40	HG-8	LL1721	MG synthase^1^	0	2.69	9.81	0.67
40	HG-8	LL1720	MG reductase^1^	1.52	3.80	6.17	1.76
40	HG-8	LL1722	Glycerol dehydrogenase	0	3.82	8.79	0.77

^1^MG = methylglyoxal.

**Figure 2:**
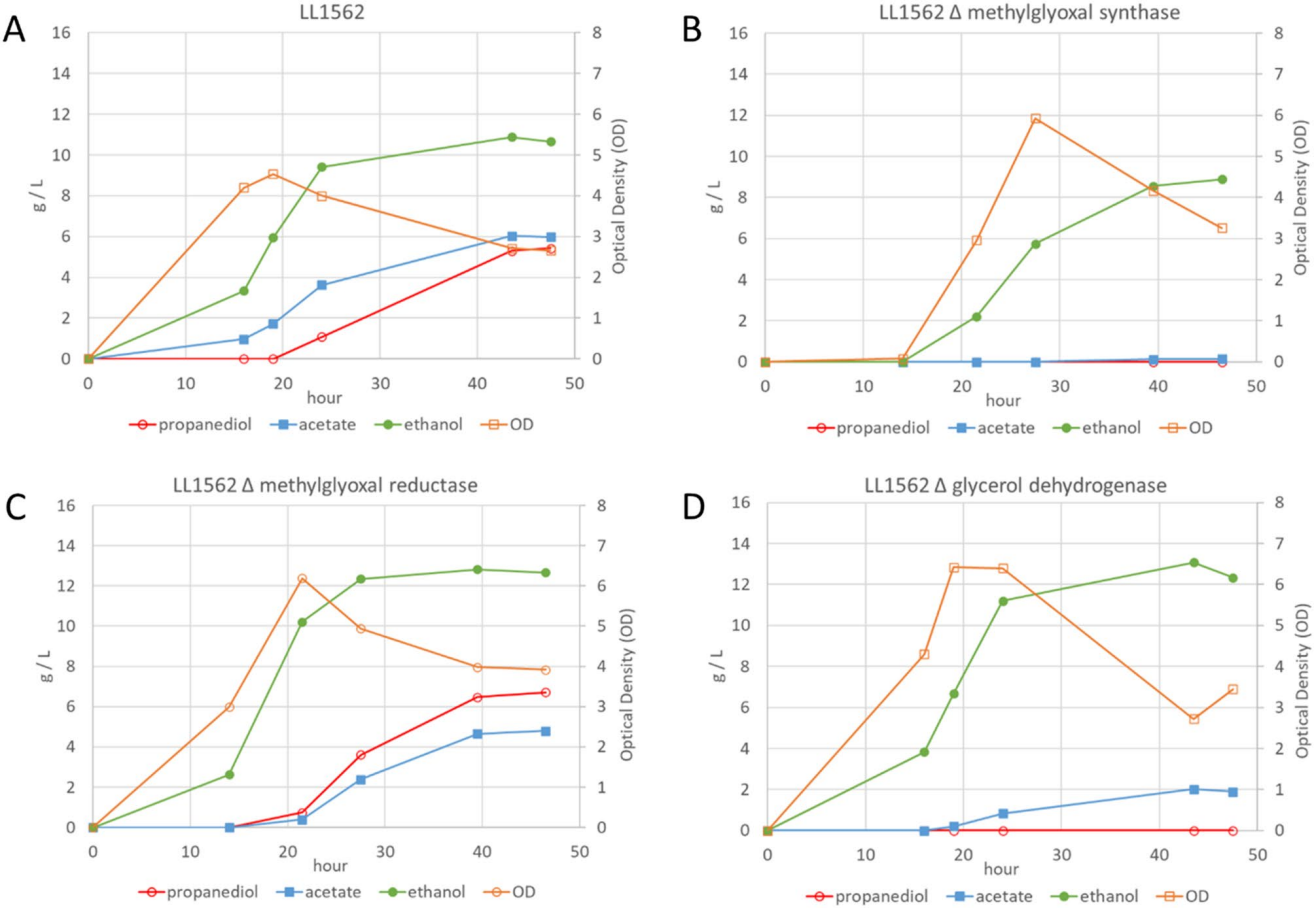
Fermentations of 60 g/L cellobiose with knockout strains in the LL1562 background, as indicated in each panel. Residual sugars (glucose and cellobiose) at the final time point were (**A**) 11.1 g/L; (**B**) 27.4 g/L; (**C**) 7.5 g/L; (**D**) 22.3 g/L.

To examine the effect of the deletions in more detail, fermentations were conducted in pH-controlled bioreactors with 60 g/L cellobiose (Fig. 2). There was no 1,2-propanediol produced in either the methylglyoxal synthase or glycerol dehydrogenase knockouts. As expected based on the pathway, hydroxyacetone accumulated in the glycerol dehydrogenase (i.e. hydroxyacetone reductase) knockout. Acetate production was very low in the methylglyoxal synthase knockout, but growth was not as robust as the other strains, with delayed maximum optical density and 27.4 g/L residual sugars (Fig. 2 legend). The methylglyoxal reductase knockout strain produced similar amounts of 1,2-propanediol, acetate and ethanol as the LL1562 parent. For each knockout, similar results were observed for the knockouts made in the wild type HG-8 strain background (data not shown).

Enzyme assays were conducted using cell free extracts prepared from two of the deletion strains made in the LL1562 ethanologen background (Table 5). Consistent with fermentation results, there was no hydroxyacetone reductase activity in the strain with a deletion of glycerol dehydrogenase. Hydroxyacetone reductase activity was observed for both NADH and NADPH. Methylglyoxal reductase activity was slightly lower in both mutants relative to the parent, but the difference was not significant. Activity was observed with NADPH but was essentially zero with NADH. As a demonstration of the potential to use the HG-8 glycerol dehydrogenase gene in other species, it was cloned and expressed in *E. coli*. Cell free extracts showed 1.34 μmol min^−1^ mg^−1^ of activity with NADH and 0.56 μmol min^−1^ mg^−1^ with NADPH, while a no-insert control showed no activity.

**Table 5:** Methylglyoxal and hydroxyacetone reductase activities in cell free extracts of deletion strains.

Specific Activity μmol min^−1^ mg^−1^	Substrate & cofactor			
	MG^1^ NADH	MG^1^ NADPH	Hydroxyacetone NADH	Hydroxyacetone NADPH
LL1562	0.07	1.55	0.91	0.57
SD, *n* = 6–16	0.06	0.49	0.22	0.12
LL1723 (LL1562 ΔMGR)	0.10	0.89	0.72	0.41
SD, *n* = 6–12	0.05	0.32	0.15	0.16
LL1725 (LL1562 ΔGDH)	0.00	0.78	0.05	0.07
SD, *n* = 6–8	0.02	0.30	0.07	0.03

^1^MG = Methylglyoxal.

These results indicate that the genes we identified for methylglyoxal synthase and glycerol dehydrogenase are required for production of 1,2-propanediol. There do not appear to be any other active genes in strain HG-8 that encode those activities. The glycerol kinase gene from HG-8 is sufficient for heterologous hydroxyacetone reductase activity in *E. coli*, and may be useful in future efforts to engineer 1,2-propanediol production at high yield. For methylglyoxal reductase, our deletions showed no obvious effect and we cannot make any conclusion about our hypothesized gene assignment. There are likely to be other genes with methylglyoxal reductase activity than those we knocked out, consistent with the broad occurrence of this activity in various other enzymes^27^.

Neither the glycerol dehydrogenase knockout nor the methylglyoxal synthase knockout are promising candidates for high ethanol-yielding strains, the former due to the accumulation of hydroxyacetone and possibly methylglyoxal, and the later due to the poor growth rate. With the objective of generating a robust ethanologen by improving the growth rate of the LL1562 methylglyoxal synthase knockout, we cultured it in a chemostat for 100 h and by serial transfer for 7 days. The resulting strain produced ethanol as the major product, but also significant amounts of acetate and lactate, the later despite the absence of lactate dehydrogenase. The observed increase in products other than ethanol in response to adaptation may be related to the programed switch to production of acetate and propanediol upon growth cessation. We would ideally generate a strain that produced ethanol in both growth and stationary phases (e.g. Figure 1D), but it is difficult to select for ethanol production in stationary phase because it is not directly growth-coupled. Elimination of methylglyoxal production may be fundamentally detrimental for strain robustness as well, due to loss of an important overflow pathway that prevents accumulation of glycolytic phosphates^28^.

### Effect of ATP on alcohol/aldehyde dehydrogenase

The observed switch between the production of ethanol and production of propanediol + acetate in strain HG-8 is remarkable, and implies that a regulatory mechanism is at play. Understanding that mechanism could be helpful for metabolic engineering efforts in this and related organisms, since control of end product distribution is a central and recurrent theme. We observed production of 1,2-propanediol to be accompanied by production of acetate, consistent with the determination of Cameron and Cooney that production of a 1:1 molar ratio is redox-balanced^14^. So whatever regulation mechanism is at work affects the production of both products simultaneously, at very different points of cellular metabolism. Control of 1,2-propanediol production could be through the regulation of methylglyoxal synthase, which is known to be tightly regulated due to the toxic properties of methylglyoxal. An example of such regulation occurs in *Bacillus subtilis*, in which the catabolite repression protein Crh binds to and inhibits methylglyoxal synthase under carbon starvation conditions^29^. But regulation of methylglyoxal synthase would not explain how acetate production increases simultaneously.

It may be noted that both ethanol and acetate are derived in two steps from the same acetyl-CoA intermediate: acetate by the action of phosphotransacetylase and acetate kinase, and ethanol by the action of a bifunctional protein comprising aldehyde dehydrogenase and alcohol dehydrogenase. A decrease in aldehyde/alcohol dehydrogenase would lead to accumulation of acetyl-coA, which could be diverted to acetate. It is also important to note that the yield of ATP from production of a 1:1 ratio of 1,2-propanediol and acetate is 1.0 mol ATP/mol glucose consumed^15^, compared to the yield of 2.0 mol ATP/mol glucose for ethanol formation. A switch from a high ATP-yielding metabolic mode to a low ATP-yielding mode upon entry into stationary phase fits with the lower ATP demands and higher need for ATP disposal in stationary phase. As cell synthesis stops, ATP levels would be expected to rise, which hypothetically could trigger the metabolic switch we observed.

A previous study in *Lactococcus lacti*s demonstrated an accumulation of ATP in resting (i.e. stationary) cells and a direct inhibition of alcohol dehydrogenase by ATP, supporting the role of ATP in regulation of product distribution^30^. To test whether ATP might play a role in the observed switching from ethanol to acetate in strain HG-8, we tested the effect of ATP addition to enzyme assays of aldehyde and alcohol dehydrogenase (Table 6). Consistent with the work of Palmfeldt et al.^30^, we observed that the addition of 10 mM ATP greatly reduced those activities in vitro. Aldehyde dehydrogenase activity (consuming acetyl-CoA and NAD(P)H) was particularly affected by ATP, being reduced to near zero. Alcohol dehydrogenase activity (consuming acetaldehyde and NAD(P)H) was higher generally, and reduced approximately 50% by the addition of ATP. Activity and inhibition was similar for cells from growth and stationary phases, indicating that changing expression of the alcohol/aldehyde dehydrogenases was not the reason for reduced flux to ethanol in stationary phase.

**Table 6:** Alcohol and aldehyde dehydrogenase activities in cell free extracts of strain HG-8.

Specific activity μmol min^−1^ mg^−1^	Substrate & cofactor							
	Acetyl-CoA NADH	+ ATP	Acetyl-CoA NADPH	+ ATP	Acetaldehyde NADH	+ ATP	Acetaldehyde NADPH	+ ATP
Growth phase cells	0.14	0.01	0.01	0.00	0.28	0.12	0.56	0.27
SD, *n* = 3	0.00	0.02	0.00	0.01	0.03	0.01	0.06	0.03
Stationary phase cells	0.11	0.02	0.02	0.00	0.34	0.23	1.22	0.59
SD, *n* = 3	0.02	0.01	0.01	0.01	0.03	0.02	0.15	0.07

Hon et al.^31^ previously showed that magnesium stimulates the activity of *T. saccharolyticum* aldehyde dehydrogenase. Since ATP is known to chelate magnesium^32^, it is possible that the observed inhibition is due to chelation of magnesium present in the cell extracts. Whether such chelation could happen following a spike in ATP levels in vivo is unknown, though a recent structural study of ATP inhibition of a human aldehyde dehydrogenase showed a complex interplay of ATP and magnesium binding^33^.

Our results support an important regulatory role for ATP in the growth phase-associated metabolic switching we observed in strain HG-8. It is yet to be seen whether ATP directly regulates the formation of 1,2-propanediol in a similar way. It will also be important to determine whether ATP levels affect the activity of aldehyde/alcohol dehydrogenase in other ethanol-producing species, such as *C. thermocellum*. Sustained ethanol formation in stationary phase is a very important trait for ethanologens, and this result provides insight into an important factor to be considered in the engineering of that trait.

The production of 1,2-propanediol *T. thermosaccharolyticum* was established decades ago, but the genes involved were not previously identified. The work described herein identifying two key genes will be useful in applied efforts to improve 1,2-propanediol production in *T. thermosaccharolyticum* or to enable its heterologous production in other organisms. The metabolic switching from ethanol production to acetate and 1,2-propanediol production is also significant and interesting from a scientific perspective for a number of reasons. While growth phase coupled metabolic mode switching has been described in *Clostridium acetobutylicum*, it has not been previously described in *Thermoanaerobacterium* to the best of our knowledge. The metabolic state of the cell is expected to differ markedly between exponential growth phase and stationary phase, especially in the biosynthetic needs for energy and for molecular precursors. The continuation of metabolism from one phase to the other would seem to require adjustment of one form or another, and the case here is an interesting and possibly instructive example. Further investigation of the mechanism of this switch, whether by increased ATP levels as we hypothesize here, or some other mechanism, may lead to improved abilities to manipulate product formation, and to extend product formation during stationary phase, a key factor in improving industrial product yield.

## Methods

### Genetic manipulations

The genome sequence for *T. thermosaccharolyticum* strain HG-8 (AKA LL1244) was generated by JGI. A draft assembly was used for the work described, but the genome sequence was recently updated and released under Accession Number GCA_024171655.1.

*Markerless gene deletions for high ethanol yield.* The initial thymidine kinase knockout LL1411 was made by transforming HG-8 with plasmid pMU2105 (Supplementary Table 1) (made by Joe Shaw, Mascoma Corp.) and selecting for resistance to 10 μg/ml 5-fluorodeoxyuridine (FUDR). Subsequently, a two step gene knockout procedure was followed to make markerless deletions, based on previous methods for *T. saccharolyticum*^34^. Two ‘suicide’ plasmids that replicate in *E. coli* but not *T. thermosaccharolyticum* were constructed for each deletion. The first plasmid carries the kanamycin resistance gene and thymidine kinase genes along with two flanking regions matching the chromosomal target. The second plasmid carries the fused flanking regions, used in counterselection (step 2). Both plasmids were constructed using Gibson assembly^35^, designing the primers with 26 bp overlap (Supplementary Table 2).

In step 1, the first suicide plasmid was transformed into *T. thermosaccharolyticum* by natural competence in M122C medium, pH 6.7 and selected for integration into the chromosome with 200 μg/ml kanamycin, deleting the targeted gene in the process. Colony PCR with primers external to the homology regions was used to identify colonies carrying the correct chromosomal insertion, which were then replated and retested by PCR. In step 2, the second suicide plasmid was transformed by natural competence, and selected on M122C medium + FUDR, which selects for loss of thymidine kinase gene. A range of different amounts of the transformed cells were plated, usually a fivefold dilution series, to assure effective counterselection. A 'no DNA' negative control transformation was included to see the background rate of spontaneous FUDR resistance, as well as 'no FUDR' controls. Colonies were screened with the same external primers, looking for deletion of both the targeted gene and the integrated kanamycin resistance/thymidine kinase genes. Candidate colonies were replated and re-tested by PCR.

#### Deletion of genes related to 1,2-propanediol

A similar method was used to delete the genes related to 1,2-propanediol production, performing step 1 of the procedure described above. The plasmids pCH50, pCH51 and pCH53, targeting the genes encoding methylglyoxal reductase, methylglyoxal synthase and glycerol dehydrogenase respectively, are shown in Supplementary Fig. 1. Colony PCR with primers external to the homology regions was used to identify colonies carrying the correct chromosomal insertion. In the case of the glycerol dehydrogenase knockout, the external primers CH309-CH310 amplified a smaller than expected product so the forward external primer CH309 was paired with primers CH308 in the thymidine kinase gene or CH321 in the glycerol dehydrogenase gene to identify the correct insertion (Supplementary Fig. 2). Candidate colonies were replated and re-tested by PCR, verifying a) expected size using external primers; b) absence of PCR product for primers in the deleted region; c) presence of homology flanking regions adjoining the deletion.

Glycerol dehydrogenase was expressed in *E. coli* by cloning into the pBAD Directional TOPO® TA Expression Kit (Invitrogen) with the primers CH351 and CH364.

### Fermentations and analysis

Medium CC6, containing 5 g/L yeast extract and other components^36^ was used in both anaerobic serum bottle fermentations of 20–50 ml liquid volume and pH-controlled fermentors of 1 L volume. Bottle fermentations also contained buffering agents, either 10 g/L calcium carbonate or 5 g/L MOPS with initial pH adjusted to 6.2 or 7.0, respectively. Serum bottles were sealed with butyl rubber stoppers and flushed with 20% CO_2_/80% N_2_ prior to autoclaving, then incubated on a shaking incubator at 55 °C after inoculation.

For Table 3 “AA” refers to Sigma # M5550 MEM amino acid solution, and “Vit” refers to Sigma # R7256 RPMI 1640 vitamin solutions. Amino acid Group A was 33 μg/ml L-phenylalanine, 52.5 μg/ml L-isoleucine, 10.2 μg/ml L-tryptophan, 46.8 μg/ml L-valine, 52.4 μg/ml L-leucine. Amino acid Group B was 126.4 μg/ml L-arginine•HCl, 31.28 μg/ml L-cystine • 2HCl, 42 μg/ml L-histidine•HCl•H2O, 72.5 μg/ml L-lysine•HCl, 15.1 μg/ml L-methionine, 47.6 μg/ml L-threonine.

Bioreactor fermentations were carried out in Biostat A-plus fermentors (Sartorius) at 55 °C and stirring at 200 rpm. Reactors containing cellobiose and water were autoclaved for 1.5 h on liquid cycle then sparged with 20% CO_2_/80% N_2_ while cooling to 55 °C. A 10 × concentrate of CC6 medium was added by filter sterilization. The pH was set to 7.0 and maintained by the addition of 4 M KOH. Gas production was monitored using Milligascounter meters from Ritter (Germany).

Fermentation products were analyzed with an Aminex HPX-87H column (Bio-Rad, Hercules, CA) at 60 °C, with RI (refractive index) detection and a 5-mM sulfuric acid solution eluent at a flow rate of 0.6 ml/min.

### Enzyme assays

Cells of strain HG-8 were grown in a bioreactor with 40 g/L cellobiose and 4 × 50 ml samples were removed after 13 and 19 h. They were centrifuged and then washed twice with 1 ml of 0.1 M potassium phosphate pH 7.1, centrifuged again and resuspended in 0.5 ml of 0.1 M potassium phosphate before being frozen at −80 °C. To prepare cell free extract, cells were thawed in an anaerobic chamber, mixed and 100 μl was diluted in 400 μl of 0.1 M potassium phosphate. 1 μl Ready-Lyse (Epicentre) was added and incubated 5–10 min at room temperature. 1 μl (5U) DNAse I (Thermo Scientific) was added and incubated 5–10 min at room temperature. Cell debris was removed by centrifugation for 6 min at 12,000 xg, then the supernatant was removed to a new tube and kept on ice for the assays.

Assays were conducted in 1.0 cm quartz cuvettes in an anaerobic chamber using an Agilent Technologies 8453 UV–visible spectrophotometer with multicell cuvette holder attached to a circulating water-bath set at 55 °C. Reactants were added to cuvettes containing 950 μl of potassium phosphate pH 7.0 and mixed by pipetting. Reagents and their final concentrations included the following: 0.4 mM NADH Sigma # N8129, 0.4 mM NADPH Sigma # N7505, 10 mM acetaldehyde Sigma # 402,788, 1 mM Acetyl-CoA sodium salt Sigma # A2056, 10 mM ATP Sigma # A2383, and either 4 μl or 8 μl of cell free extract. Reactions containing NADH were started by the addition of either acetaldehyde or acetyl-CoA, but this was not possible for NADPH, due to high background activity. For reactions with NADPH, they were started by addition of cell free extract instead. NAD(P)H concentration was monitored at 340 nm using an extinction coefficient of 6.3 mM^−1^ cm^−1^. Protein concentrations of cell free extracts were determined by Bradford assay using Pierce Coomassie Plus reagent (Thermo Fisher Scientific).

## Supplementary Information

Supplementary Information.

## Data Availability

The datasets generated during and/or analyzed during the current study are available from the corresponding author on reasonable request. The DNA sequence data are available in the GenBank repository, Accession #s OP763091—OP763099.
