# Supplementary material for: Growth-uncoupled propanediol production in a Thermoanaerobacterium thermosaccharolyticum strain engineered for high ethanol yield
Source: Sci Rep. 2023 Feb 10;13:2394. doi: 10.1038/s41598-023-29220-9 (PMC9918460; doi:10.1038/s41598-023-29220-9)
Supplement: Supplementary file 1 — Supplementary Information. [file 41598_2023_29220_MOESM1_ESM.docx]

**Supplementary Information** for Herring et al. Growth-uncoupled propanediol production in a *Thermoanaerobacterium thermosaccharolyticum* strain engineered for high ethanol yield

**Supplementary Table 1.** Plasmids used.

| **Plasmid** | **GenBank Accession** | **Description** | **PCR products (primer numbers)** |
| --- | --- | --- | --- |
| pMU2105 | OP763091 | Deletion of *tdk* |  |
| pCH13 | OP763092 | Insert kan-tdk in *pta* | CH45-46, CH57-58, CH59-60, CH65-30, CH31-67 |
| pCH16 | OP763093 | Insert kan-tdk in *ldh* | CH45-46, CH76-96, CH79-97, CH98-99 |
| pCH19 | OP763094 | Insert kan-tdk in *lctD* | CH45-46, CH125-126, CH127-128, CH131-132 |
| pCH22 | OP763095 | Insert kan-tdk in *rnf* | CH45-46, CH143-144, CH145-146, CH149-150 |
| pCH24 | OP763096 | Insert kan-tdk in *hfsB* | CH45-46, CH157-158, CH159-160, CH163-164 |
| pCH50 | OP763097 | Insert kan-tdk into MG reductase | CH45-46, CH287-288, CH289-290, CH291-292 |
| pCH51 | OP763098 | Insert kan-tdk into MG synthase | CH45-46, CH295-296, CH297-298, CH299-300 |
| pCH53 | OP763099 | Insert kan-tdk into glycerol DH | CH45-46, CH303-304, CH305-313, CH307-308 |

**Supplementary Table 2.** Oligonucleotide primers.

| **Primer number** | **Description** | **Sequence** | **Product size** |
| --- | --- | --- | --- |
| CH30 | Kan region rev | tcattattttgcaAAAGCTTTTTAGACATCTAAATCTAGGTAC |  |
| CH31 | tdk gene&promoter fwd | tctaaaaagctttTGCAAAATAATGAAGTGGTTAG |  |
| CH45 | pUC19@bp633 fwd | ACGTCTAAGAAACCATTATTATCATgcattaatgaatcggccaac | 1989 bp |
| CH46 | pUC19@bp2617 rev | acgtcaggtggcacttttcggggaa | 1989 bp |
| CH49 | pta/ack external X09592 | tcttcttattctgctttctgtgtacct |  |
| CH50 | pta/ack external X09593 | AATGCCATATCAGGTGTCGGCTCA |  |
| CH51 | tdk external thermosac | CCTTACTTGCTCCTAATGTAAACGA | 2416 bp |
| CH52 | tdk external thermosac | TTATCTGGTGGCCTTGATCC | 2416 bp |
| CH57 | pta up fwd | ttccccgaaaagtgccacctgacgtTGACAGAGCTATTTCACGTGCT |  |
| CH58 | pta up rev | gggtttatcgaccGCTGGCCTTAACAAATCTGC |  |
| CH59 | tween genes down fwd | tcctcgtacgtagAGGGTAGTGCATCCCATGAA |  |
| CH60 | tween genes down rev | atgataataatggtttcttagacgtGCTGCTCTTTGCGAAACATA |  |
| CH65 | Kan region fwd pair to CH58 | tgttaaggccagcGGTCGATAAACCCAGCGAAC |  |
| CH67 | tdk rev pair to CH59 | gatgcactaccctCTACGTACGAGGAACTTC |  |
| CH71 | pta up rev | ccttgcgttattgTCATAAAATCGTCCCTTCCCTA |  |
| CH72 | tween genes | gacgattttatgaCAATAACGCAAGGAATTGGTG |  |
| CH73 | pta up rev | cctttaactgccgGCTGGCCTTAACAAATCTGC |  |
| CH74 | ack small down | tgttaaggccagcCGGCAGTTAAAGGTGGAAAA |  |
| CH75 | ack small down | atgataataatggtttcttagacgtCTCATTAGTCGGCACAACCA |  |
| CH76 | ldh up fwd | ttccccgaaaagtgccacctgacgtTCGCTCATTTTAAATATCAATCCTT |  |
| CH77 | ldh up rev | gggtttatcgaccTCTTATTTTATCCTTCTTGCTTTGAA |  |
| CH78 | ldh down fwd | tcctcgtacgtagAGCGGCTTTTATCTGAATGG |  |
| CH79 | ldh down rev | atgataataatggtttcttagacgtTCTGATGATTCCCTGTTGCT |  |
| CH80 | Kan fwd | ggataaaataagaGGTCGATAAACCCAGCGAAC |  |
| CH81 | Tdk rev | gataaaagccgctCTACGTACGAGGAACTTC |  |
| CH82 | ldh external | CCTTATCCCTTTCGCTTTCA |  |
| CH83 | ldh external | TTGGGATCGAATTTGCCTAT |  |
| CH96 | Kan rev | ggtaaaagcatgaAAAGCTTTTTAGACATCTAAATCTAGGTAC |  |
| CH97 | Tdk coding fwd | aggaggacgaaagATGTACGGTCCTAAAGACCATGG |  |
| CH98 | Ctherm CBP promoter fwd | tctaaaaagctttTCATGCTTTTACCCCTTTCA | 573 bp |
| CH99 | Ctherm CBP promoter rev | taggaccgtacatCTTTCGTCCTCCTTAAAATTTTC | 573 bp |
| CH113 | ldh up, alt 2 fwd | ttccccgaaaagtgccacctgacgtTGAACCTAATGGCAATATAGTCAGG | 973 bp |
| CH114 | ldh up, alt 2 rev | cagccacagcgtaATCAACAGGATTTGTTACAATCAG | 973 bp |
| CH115 | ldh down, alt 2 fwd | aaatcctgttgatTACGCTGTGGCTGTAGCAGT | 980 bp |
| CH116 | ldh down, alt 2 rev | atgataataatggtttcttagacgtCCAGCGGATCATTTATTGGT | 980 bp |
| CH125 | lctD up fwd | ttccccgaaaagtgccacctgacgtCGCAAGAGGAGCTGATAAGG | 878 bp |
| CH126 | lctD up rev | gggtttatcgaccTTCATCTACGATTTCAGGTGTCA | 878 bp |
| CH127 | lctD down fwd | tcctcgtacgtagGAGCCAGCACAAATCGAAGT | 922 bp |
| CH128 | lctD down rev | atgataataatggtttcttagacgtACCGTTGCAATCTCGCTTAC | 922 bp |
| CH129 | lctD external fwd | ACACGGTGAATTGAGCACAG | 3382 bp |
| CH130 | lctD external rev | TCCATGACCATCAGCACATT | 3382 bp |
| CH131 | Kan fwd | aatcgtagatgaaGGTCGATAAACCCAGCGAAC |  |
| CH132 | tdk rev | tttgtgctggctcCTACGTACGAGGAACTTC |  |
| CH133 | lctd up rev | tttgtgctggctcTTCATCTACGATTTCAGGTGTCA |  |
| CH134 | lctD down fwd | aatcgtagatgaaGAGCCAGCACAAATCGAAGT |  |
| CH143 | rnfC up fwd | ttccccgaaaagtgccacctgacgtGGCATATCGACTGCTTGCTT | 896 bp |
| CH144 | rnfC up rev | gggtttatcgaccTCGGATGTATTCCACCGAAA | 896 bp |
| CH145 | rnf down fwd | tcctcgtacgtagCGGGACTGTTATTTACGGCTA | 959 bp |
| CH146 | rnf down rev | atgataataatggtttcttagacgtGATTCTGGCGGTGATCCTAC | 959 bp |
| CH147 | rnf external | GGATGGTGATCAAATGGAAGTT | 7300 bp |
| CH148 | rnf external | GCGGTACAATAGTCGGTCCA | 7300 bp |
| CH149 | Kan fwd | ggaatacatccgaGGTCGATAAACCCAGCGAAC |  |
| CH150 | tdk rev | aataacagtcccgCTACGTACGAGGAACTTC |  |
| CH151 | rnfC up rev | aataacagtcccgTCGGATGTATTCCACCGAAA |  |
| CH152 | rnf down fwd | ggaatacatccgaCGGGACTGTTATTTACGGCTA |  |
| CH157 | hfsB up fwd | ttccccgaaaagtgccacctgacgtATAGCAGCAGTGAGGGCTTC | 866 bp |
| CH158 | hfsB up rev | gggtttatcgaccGCAGTTTCTGCAATTTGCTTC | 866 bp |
| CH159 | hfsb down fwd | tcctcgtacgtagTGCTATTAAATCAAGGTGATGATGA | 873 bp |
| CH160 | hfsB down rev | atgataataatggtttcttagacgtGGTGGTGGTATATCAGGATCG | 873 bp |
| CH161 | hfsB external | AAGGGCATATAGCTGGAATTGA | 3597 bp |
| CH162 | hfsB external | AAATAGGCGAGTTGCAGCAT | 3597 bp |
| CH163 | Kan fwd | ttgcagaaactgcGGTCGATAAACCCAGCGAAC |  |
| CH164 | tdk rev | tgatttaatagcaCTACGTACGAGGAACTTC |  |
| CH165 | hfsB flank fusion primers | tgatttaatagcaGCAGTTTCTGCAATTTGCTTC |  |
| CH166 | hfsB flank fusion primers | ttgcagaaactgcTGCTATTAAATCAAGGTGATGATGA |  |
| CH171 | Tsac adhE up | ttccccgaaaagtgccacctgacgtCGGCTGCTACTTCTTAAACAAAG | 1851 bp |
| CH172 | Tsac adhE up | gggtttatcgaccTGCTGTATAAGATTTTGATGTATGTA | 1851 bp |
| CH173 | Tsac adhE down | tcctcgtacgtagAAACTAAAGCGCCGACATTT | 937 bp |
| CH174 | Tsac adhE down | atgataataatggtttcttagacgtATGGTGGCAGCAGGATAAAG | 937 bp |
| CH175 | Kan fwd | atcttatacagcaGGTCGATAAACCCAGCGAAC |  |
| CH176 | Tdk rev | ggcgctttagtttCTACGTACGAGGAACTTC |  |
| CH177 | Tsac adhE external | GAGGGCTGTATCGGATCTCA | 2994 bp |
| CH178 | Tsac adhE external | GAAATGCTTCCAGCCCACTA | 2994 bp |
| CH287 | MG reductase KO up fwd | ttccccgaaaagtgccacctgacgtGACTGGCGAGTTGACGAAGT | 973 bp |
| CH288 | MG reductase KO up rev | gggtttatcgaccTTCCATTTTACTTTTCCTCCTCA | 973 bp |
| CH289 | MG reductase KO down fwd | tcctcgtacgtagCAGATTTGCTCAAAGATGTTGC | 997 bp |
| CH290 | MG reductase KO down rev | atgataataatggtttcttagacgtTCAAGCGCTCTAAAGTTTTATTCA | 997 bp |
| CH291 | Kan fwd | aagtaaaatggaaGGTCGATAAACCCAGCGAAC | 2540 bp |
| CH292 | tdk rev | ttgagcaaatctgCTACGTACGAGGAACTTC | 2540 bp |
| CH293 | MG reductase KO external fwd | GAATGAAAAGCCATCCGGTA | 3910 bp |
| CH294 | MG reductase KO external rev | GCCAAAGTCATATCCTTTCCA | 3910 bp |
| CH295 | MG synthase KO up fwd | ttccccgaaaagtgccacctgacgtAGCAGTTTTGGTTGGAATGAT | 991bp |
| CH296 | MG synthase KO up rev | gggtttatcgaccCAATTAATGCAATATTCAAATTAACCA | 991bp |
| CH297 | MG synthase KO down fwd | tcctcgtacgtagTGCTGGGCTTTTAGAATGGA | 988bp |
| CH298 | MG synthase KO down rev | atgataataatggtttcttagacgtCATTTAGCTTTTTGGCAACG | 988bp |
| CH299 | Kan fwd | attgcattaattgGGTCGATAAACCCAGCGAAC | 2540 bp |
| CH300 | tdk rev | taaaagcccagcaCTACGTACGAGGAACTTC | 2540 bp |
| CH301 | MG synthase KO external fwd | ATGCAGTAGAAACGGGATCG | 2412bp |
| CH302 | MG synthase KO external rev | AATTGCAGCTCCACCAAAAG | 2412bp |
| CH303 | Glycerol DH up fwd | ttccccgaaaagtgccacctgacgtCAGGAAGTGATTCCAGTCTCG | 977bp |
| CH304 | Glycerol DH up rev | gggtttatcgaccCCATTGCCTTGTACATATTTCG | 977bp |
| CH305 | Glycerol DH KO down fwd | tcctcgtacgtagTGATGCGATAGGGAAAATGT | 273 bp |
| CH307 | Kan fwd, homol to CH304 | tacaaggcaatggGGTCGATAAACCCAGCGAAC | 2540 bp |
| CH308 | tdk rev, homol to CH305 | ccctatcgcatcaCTACGTACGAGGAACTTC | 2540 bp |
| CH309 | Glycerol DH KO external fwd | ATAATGCGGCAAAAAGAGGA | 3279bp |
| CH310 | Glycerol DH KO external rev | CATGTCCAAGAACAGCTGGA | 3279bp |
| CH313 | Glycerol DH KO down rev | atgataataatggtttcttagacgtTTTTAGTTTGCCATGGATGAAA | 273 bp |
| CH321 | Glycerol DH internal rev | GGTCGCCAATTATTAGCACAT |  |
| CH330 | MG reductase ORF fwd | GCCGTGCGATAAAGAACAAT | 546 bp |
| CH331 | MG reductase ORF rev | GCCTCTCTGGCTGCTATTTC | 546 bp |
| CH332 | MG synthase ORF fwd | CACAGGTGCTACAGGCCAAT | 208 bp |
| CH333 | MG synthase ORF rev | TGAATGAACATCGCATACCC | 208 bp |
| CH334 | GlycDH ORF fwd | TGAATCATTTGGCGGAGAAT | 676 bp |
| CH335 | GlycDH ORF rev | TTCAAGCACCAACTGTACCAA | 676 bp |
| CH351 | Glycerol DH coding region | GGTCGCCAATTATTAGCACAT | 1136 bp |
| CH364 | Glycerol DH coding region, TOPO | caccATGACAAAAGCTATAATAGGCCCTTC | 1107 bp |


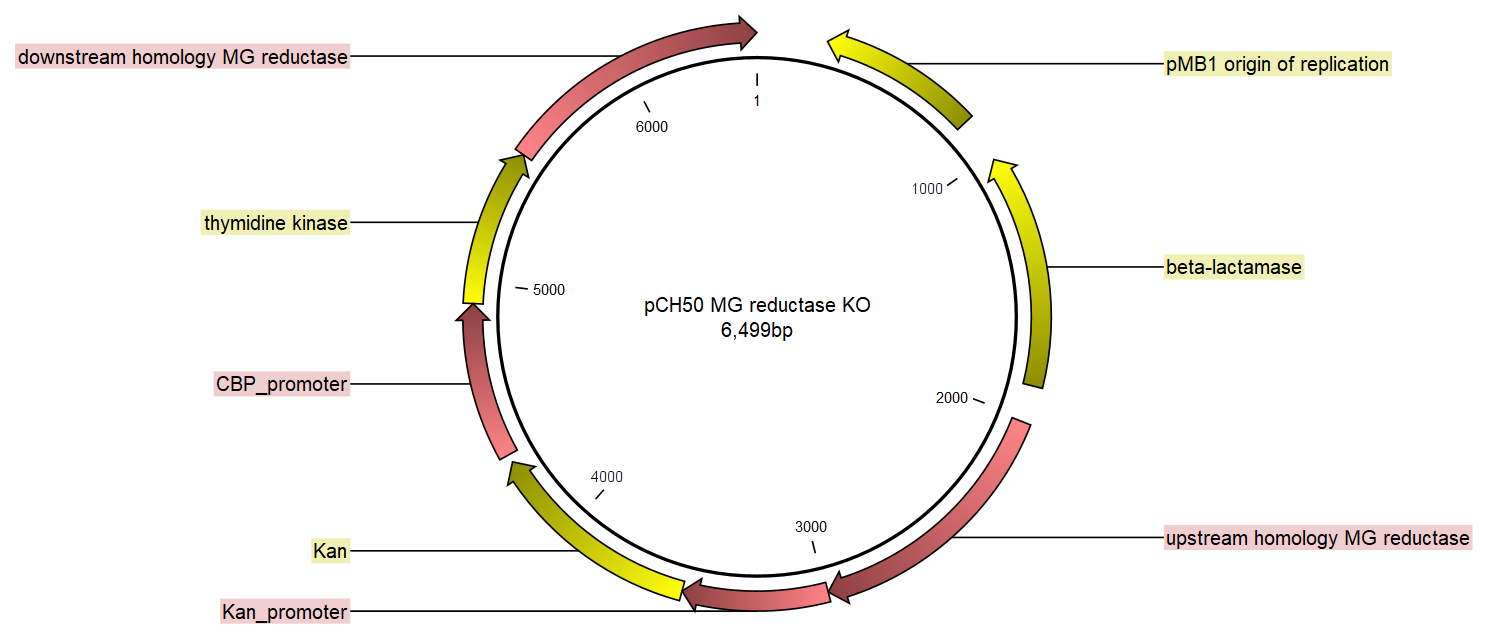

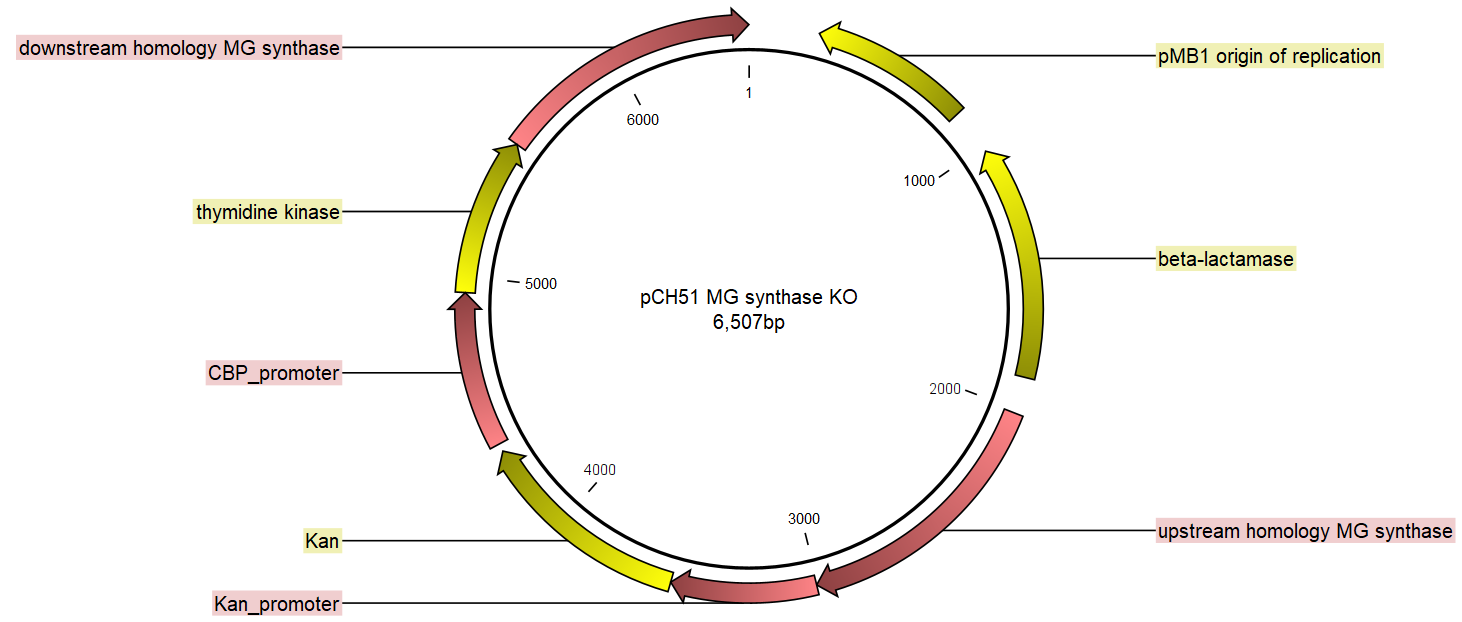

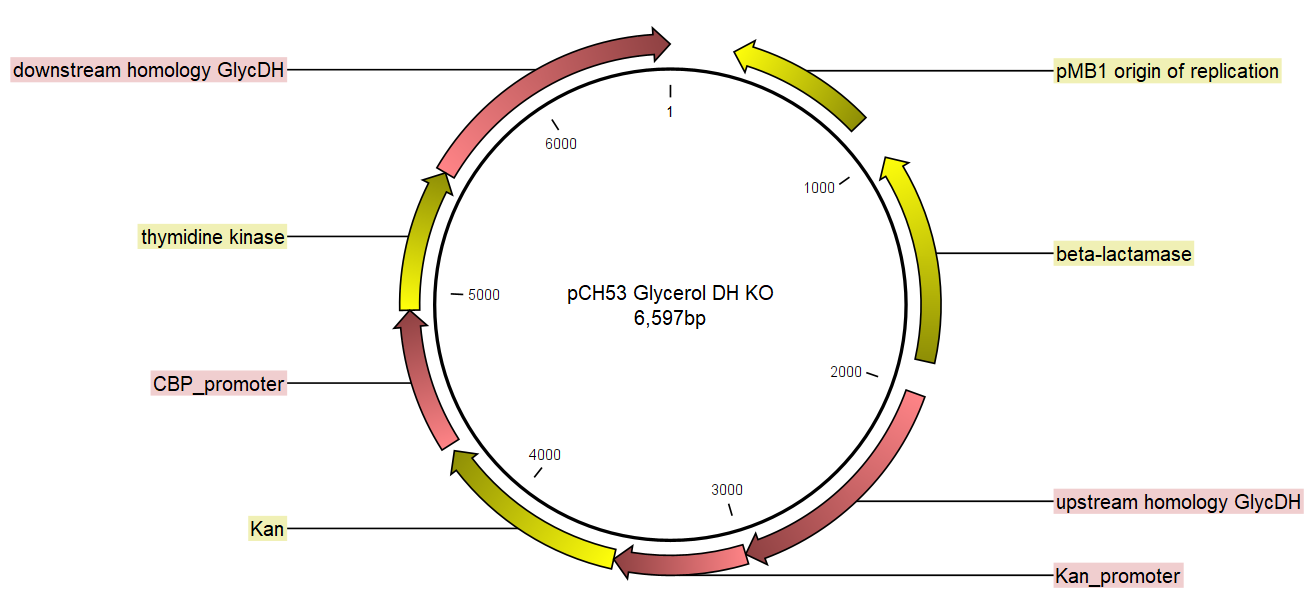


**Supplementary Figure 1.** Plasmids used to delete genes related to 1,2-propanediol


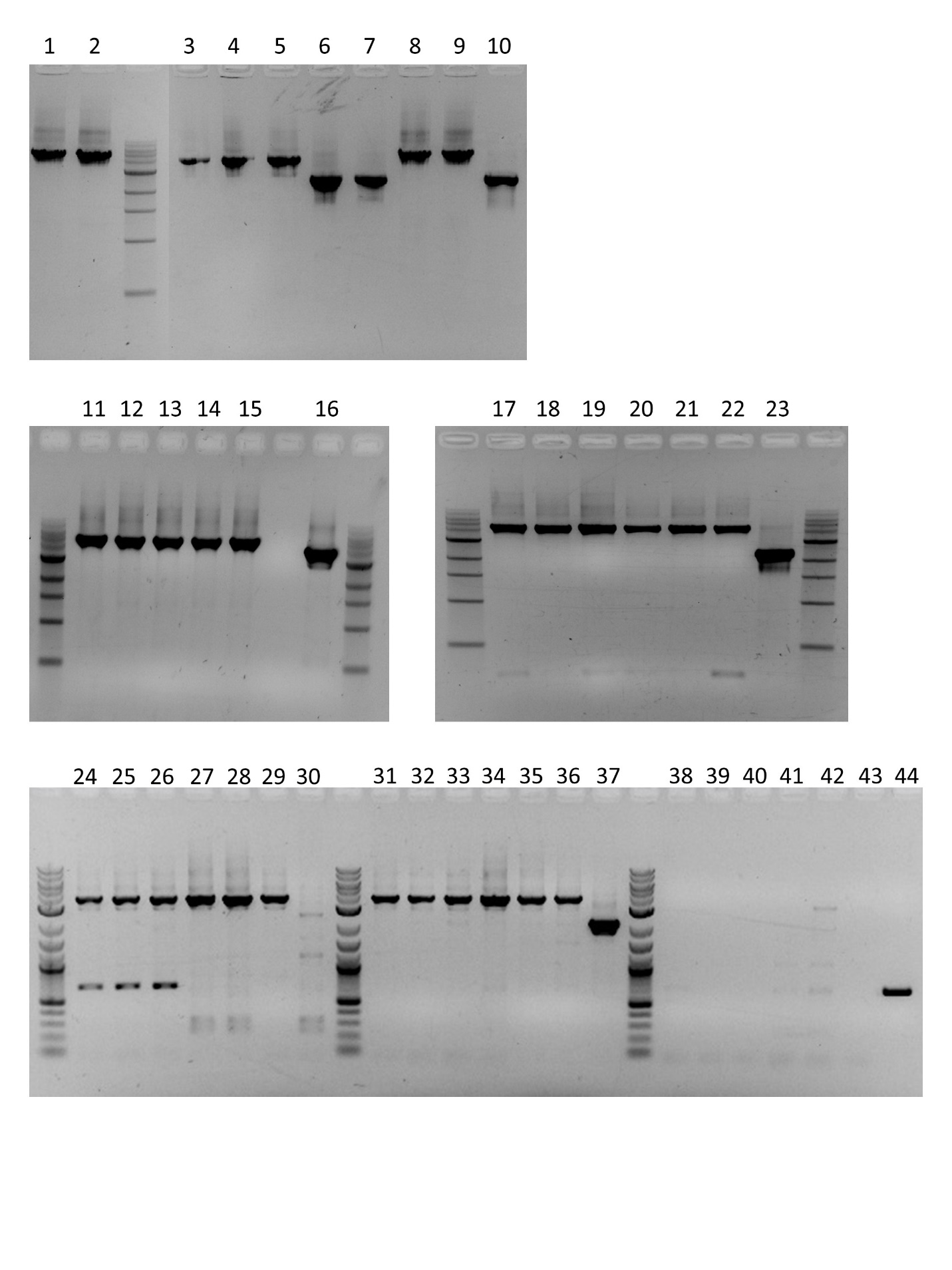


**Supplementary Figure 2.** PCR confirmation of gene deletions related to 1,2-propanediol. Lanes numbered as per Supplementary Table 3.

**Supplementary Table 3.** Legend for PCR in Supplementary Figure 2.

| Lane | Template | Primers | Primer Description |
| --- | --- | --- | --- |
| 1 | Replated colony, LL1562 + pCH50 | CH293-CH294 | MG reductase external |
| 2 | Replated colony, LL1562 + pCH50 | CH293-CH294 | MG reductase external |
| 3 | Replated colony, LL1562 + pCH51 | CH293-CH294 | MG reductase external |
| 4 | Replated colony, LL1562 + pCH51 | CH293-CH294 | MG reductase external |
| 5 | WT gDNA | CH293-CH294 | MG reductase external |
| 6 | Replated colony, LL1562 + pCH50 | CH301-CH302 | MG synthase external |
| 7 | Replated colony, LL1562 + pCH50 | CH301-CH302 | MG synthase external |
| 8 | Replated colony, LL1562 + pCH51 | CH301-CH302 | MG synthase external |
| 9 | Replated colony, LL1562 + pCH51 | CH301-CH302 | MG synthase external |
| 10 | WT gDNA | CH301-CH302 | MG synthase external |
| 11 | Replated colony, LL1244 + pCH50 | CH293-CH294 | MG reductase external |
| 12 | Replated colony, LL1244 + pCH50 | CH293-CH294 | MG reductase external |
| 13 | Replated colony, LL1244 + pCH50 | CH293-CH294 | MG reductase external |
| 14 | Replated colony, LL1244 + pCH50 | CH293-CH294 | MG reductase external |
| 15 | Replated colony, LL1244 + pCH50 | CH293-CH294 | MG reductase external |
| 16 | WT gDNA | CH293-CH294 | MG reductase external |
| 17 | Replated colony, LL1244 + pCH51 | CH301-CH302 | MG synthase external |
| 18 | Replated colony, LL1244 + pCH51 | CH301-CH302 | MG synthase external |
| 19 | Replated colony, LL1244 + pCH51 | CH301-CH302 | MG synthase external |
| 20 | Replated colony, LL1244 + pCH51 | CH301-CH302 | MG synthase external |
| 21 | Replated colony, LL1244 + pCH51 | CH301-CH302 | MG synthase external |
| 22 | Replated colony, LL1244 + pCH51 | CH301-CH302 | MG synthase external |
| 23 | WT gDNA | CH301-CH302 | MG synthase external |
| 24 | Replated colony, LL1562 + pCH53 | CH309-CH308 | GlycDH external fwd - tdk rev |
| 25 | Replated colony, LL1562 + pCH53 | CH309-CH308 | GlycDH external fwd - tdk rev |
| 26 | Replated colony, LL1562 + pCH53 | CH309-CH308 | GlycDH external fwd - tdk rev |
| 27 | Replated colony, LL1244 + pCH53 | CH309-CH308 | GlycDH external fwd - tdk rev |
| 28 | Replated colony, LL1244 + pCH53 | CH309-CH308 | GlycDH external fwd - tdk rev |
| 29 | Replated colony, LL1244 + pCH53 | CH309-CH308 | GlycDH external fwd - tdk rev |
| 30 | WT gDNA | CH309-CH308 | GlycDH external fwd - tdk rev |
| 31 | Replated colony, LL1562 + pCH53 | CH309-CH321 | GlycDH external fwd - GlycDH CDS rev |
| 32 | Replated colony, LL1562 + pCH53 | CH309-CH321 | GlycDH external fwd - GlycDH CDS rev |
| 33 | Replated colony, LL1562 + pCH53 | CH309-CH321 | GlycDH external fwd - GlycDH CDS rev |
| 34 | Replated colony, LL1244 + pCH53 | CH309-CH321 | GlycDH external fwd - GlycDH CDS rev |
| 35 | Replated colony, LL1244 + pCH53 | CH309-CH321 | GlycDH external fwd - GlycDH CDS rev |
| 36 | Replated colony, LL1244 + pCH53 | CH309-CH321 | GlycDH external fwd - GlycDH CDS rev |
| 37 | WT gDNA | CH309-CH321 | GlycDH external fwd - GlycDH CDS rev |
| 38 | Replated colony, LL1562 + pCH53 | CH334-335 | GlycDH CDS |
| 39 | Replated colony, LL1562 + pCH53 | CH334-335 | GlycDH CDS |
| 40 | Replated colony, LL1562 + pCH53 | CH334-335 | GlycDH CDS |
| 41 | Replated colony, LL1244 + pCH53 | CH334-335 | GlycDH CDS |
| 42 | Replated colony, LL1244 + pCH53 | CH334-335 | GlycDH CDS |
| 43 | Replated colony, LL1244 + pCH53 | CH334-335 | GlycDH CDS |
| 44 | WT gDNA | CH334-335 | GlycDH CDS |
